# Microvascular Changes Associated with Optic Disc Drusen: Case Report

**DOI:** 10.4274/tjo.galenos.2019.14194

**Published:** 2019-10-24

**Authors:** Özlem Biçer, Huban Atilla

**Affiliations:** 1 Ankara University Faculty of Medicine, Department of Ophthalmology, Ankara, Turkey

**Keywords:** Optic disc drusen, optical coherence tomography angiography, imaging modalities, pseudopapilledema, microvascular changes

## Abstract

Optic disc drusen (ODD) is an important clinical entity that is sometimes misdiagnosed as papilledema because of elevated and blurred disc margins. A 17-year-old male who presented with headaches underwent detailed ophthalmological examination as well as colored fundus photography, B-scan ultrasonography (USG), fundus autofluorescence (FAF), optical coherence tomography (OCT), optical coherence tomography angiography (OCTA), and visual field testing. His visual acuity was 10/10 in both eyes. Fundus examination revealed bilateral blurred and elevated optic disc margins. Diagnosis of bilateral ODD was confirmed with B-scan USG. FAF imaging revealed hyperautofluorescent areas on both optic discs. Optic nerve head OCT scans showed elevated irregular disc borders and thinning of the retinal nerve fiber layer in both eyes. On visual field testing, loss of the nasal visual field was detected in the left eye. OCTA imaging showed focal capillary dropout, especially in the nasal peripapillary area, in both eyes and reduced peripapillary and macular vessel density. In this case report, we evaluated the clinical findings and the structural features of bilateral ODD with multimodal imaging modalities including OCTA.

## Introduction

Pseudopapilledema is an abnormal and elevated appearance of the optic nerve head that is not associated with increased intracranial pressure or edema in the nerve fiber layer. Optic disc drusen (ODD) is the leading cause of this condition.^1^ The prevalence of ODD in the population is between 3.4 and 24 per 1000 according to clinical studies, while the rate is 1-2.4% in histological examinations.^2^ It is more common in females and usually bilateral.^3,4^ In most patients, it is not associated with any ocular or systemic disease and is detected incidentally during routine examination.^4^

Superficial ODD are easily recognizable by the presence of yellow hyaline-like deposits on ophthalmoscopic examination. However, because deep or buried ODD (which are more common in children especially) are not visible during examination, additional imaging methods are needed to differentiate from more serious conditions such as increased intracranial pressure and tumors.^5^ A wide variety of imaging methods are used for diagnosis, including ultrasonography (USG), fundus autofluorescence (FAF), optical coherence tomography (OCT), fluorescein angiography (FA), and computed tomography (CT).^6,7^ With the recent introduction of optical coherence tomography angiography (OCTA), peripapillary and macular retinal vessels can now be visualized noninvasively without the use of contrast agents. This provides more detailed information about optic nerve head perfusion.^8^

In this case report, we aimed to present the clinical features of a patient with ODD and the diagnostic methods used.

## Case Report

**Figure 1:**
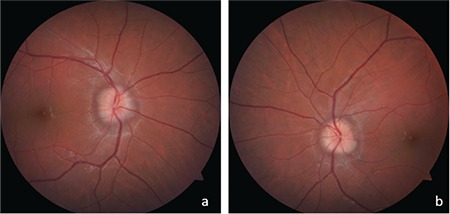
Optic disc swelling and blurred disc margins in the right (a) and left (b) eyes

A 17-year-old male presented with a 2-month history of headaches. The patient had no known systemic disease or history of trauma, drug use, or smoking. His family history was unremarkable, with no consanguinity. On ophthalmologic examination, his uncorrected visual acuity was 10/10 (0.0 LogMAR) in both eyes. His pupils were isochoric and light reflexes were normal in both eyes; no afferent pupillary defect was observed. Color vision test using Ishihara cards was normal in both eyes. Slit-lamp anterior segment examination was also normal. Intraocular pressure was 14 mmHg in the right eye and 16 mmHg in the left eye. Fundus examination revealed bilateral optic disc swelling and blurred disc margins (Figures 1a, b).

**Figure 2:**
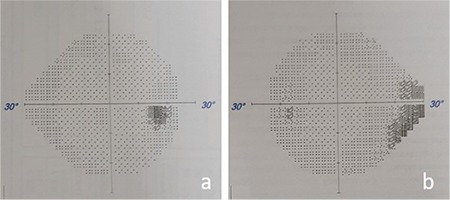
Visual fields of the right (a) and left (b) eyes demontrate unilateral scotoma involving the nasal area

**Figure 3:**
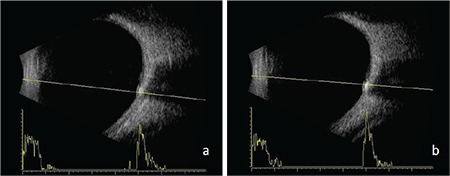
Hyperechogenic appearance on the papilla on ultrasonography in the right (a) and left (b) eyes

**Figure 4:**
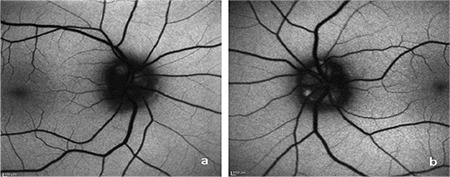
Oval-shaped drusen showing disc hyperautofluorescence on fundus autofluorescence imaging in the right (a) and left (b) eyes

**Figure 5:**
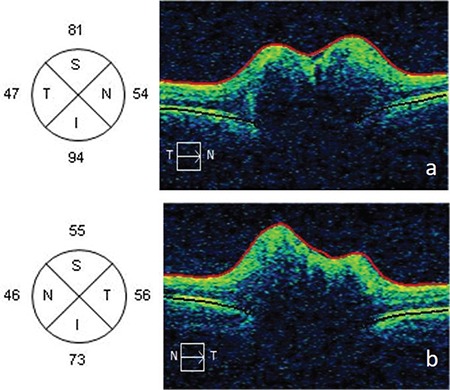
Optic disc nerve head swelling and retinal nerve fiber layer analysis on optical coherence tomography of the right (a) and left (b) eyes

**Figure 6:**
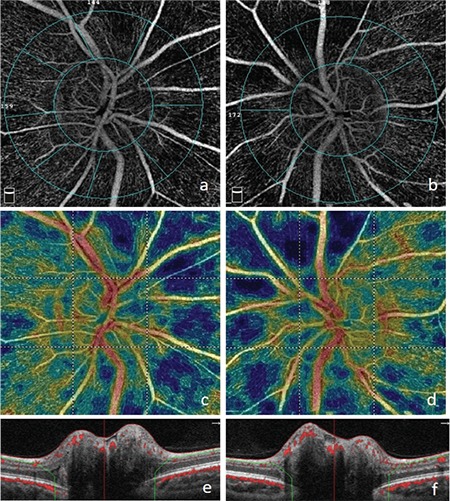
Nonperfusion areas in the radial peripapillary capillaries on optical coherence tomography angiography in the right (a) and left (b) eyes; reduced vascular density consistent with the blue areas on color vascular density map in the right (c) and left (d) eyes; B-scan optical coherence tomography images of the right (e) and left (f) eyes

**Figure 7:**
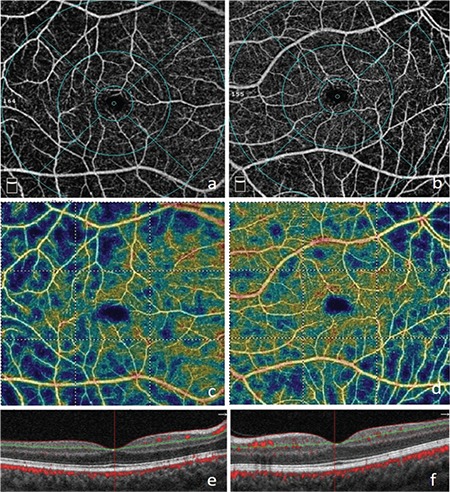
Nonperfusion areas in the macular superficial capillary plexus layer on optical coherence tomography angiography in the right (a) and left (b) eyes; reduced vascular density consistent with the blue areas on the color vascular density map in the right (c) and left (d) eyes; B-scan optical coherence tomography images of the right (e) and left (f) eyes

**Figure 8:**
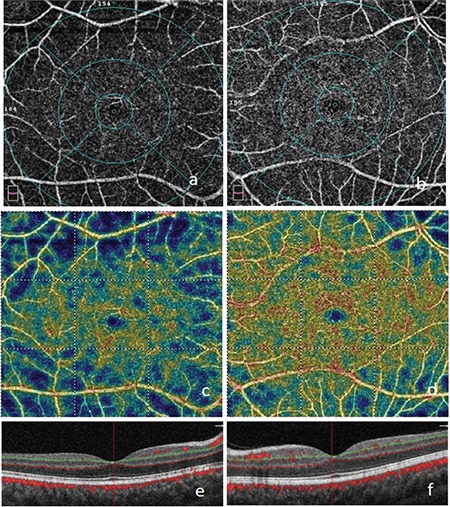
Nonperfusion areas in the macular deep capillary plexus layer on optical coherence tomography angiography in the right (a) and left (b) eyes; reduced vascular density consistent with the blue areas on the color vascular density map in the right (c) and left (d) eyes; B-scan optical coherence tomography images of the right (e) and left (f) eyes

Visual field was evaluated using the Swedish Interactive Thresholding Algorithm (SITA) standard 24-2 threshold test on a Humphrey Field Analyzer III 750 (Zeiss Humphrey Systems) automated perimetry device. Scotoma was not detected in the right eye, while a significant visual field defect was evident in the inferonasal quadrant in the left eye (Figures 2a, b). B-mode USG (AVISO, Quantel Medical, Clermont-Ferrand, France) revealed a hyperechogenic appearance consistent with bilateral ODD on the papilla (Figures 3a, b). FAF (Heidelberg Retinal Angiography 2, Heidelberg, Germany) imaging revealed oval hyperautofluorescent areas on the optic disc that were more prominent in the left eye (Figures 4a, b). On spectral domain OCT (Cirrus, Carl Zeiss Meditec Inc., Dublin, CA, USA), the mean retinal nerve fiber layer (RNFL) thickness was 69 µm in the right eye and 57 µm in the left eye despite the bilateral optic disc head swelling (Figure 5a, b). OCTA (RTVue XR ‘Avanti’, Optovue, Fremont, California, USA) of the optic disc revealed areas of capillary dropout in the retinal peripapillary layer that were more prominent in the nasal quadrant and reduced vascular density in both eyes (Figure 6a-f). Macular OCTA revealed a decrease in vascular density suggesting ischemia in the superficial and deep capillary plexus layers bilaterally (Figure 7a-f, Figure 8a-f).

## Discussion

The prevalence of ODD is reported to be 0.2-2% in adults and 0.37-1% in children. The lower than expected prevalence in children has been attributed to difficulty in the use of imaging techniques in the diagnosis of deeply buried non-calcified drusen.^9^ ODD are typically deeply situated in very young children and may eventually become superficial in late childhood, around 12 years of age.^10^

Although the pathogenesis is not known, drusen are believed to directly damage the retinal nerve fibers by axonal compression and indirectly cause ischemia in the nerve fiber layer as a result of vascular compression.^11^ It has been reported in the literature that vascular complications such as nonarteritic anterior ischemic optic neuropathy, choroidal neovascularization, and central retinal artery and vein occlusions may occur due to ODD, albeit rarely.^12^

Kovarik et al.^13^ reported that 76% of children presenting with suspected papilledema had pseudopapilledema. Misdiagnosis leads to unnecessary radiological imaging and invasive and expensive tests such as lumbar puncture or magnetic resonance imaging. Therefore, it is extremely important to be able to differentiate pseudopapilledema from papilledema, which has very different treatment, follow-up, and diagnosis. Fundus examination findings in favor of ODD are an absence of dilated capillary vessels over the disc, no blurring of the vessels around the disc, a nonhyperemic disc, and the absence of peripapillary RNFL thickening.^4^ Additional imaging methods are often needed to confirm the diagnosis.

B-mode USG remains the most reliable method for diagnosing ODD. While FAF imaging is useful for the diagnosis of superficial drusen, it can detect only 12-27% of buried drusen. OCT provides objective data through quantitative evaluation of the RNFL. Peripapillary RNFL thickness was reported to be greater in patients with papilledema compared to ODD. While RNFL values are often normal in patients with buried ODD, peripapillary thinning is observed in all quadrants in cases of superficial ODD.^2,7,9^ In accordance with literature, OCT revealed peripapillary RNFL thinning in our patient.

Visual field defects have been detected in 73% of superficial drusen cases, compared to only 36% for buried drusen.^14^ Visual field defects are less common in children (11-51%) than adults (50-90%). In patients with ODD, visual field defects have been related to older age, vision loss, and superficial, calcified drusen.^9^ In children, the most common visual field problems associated with ODD are nasal defects (54%), concentric narrowing (21%), and blind spot enlargement (18%). In addition, defects were reported to be more common in the inferonasal retinal nerve fiber bundles than superotemporal.^15^ Gaier et al.^16^ detected inferotemporal microvascular attenuation in OCTA consistent with a visual field defect in the superonasal quadrant in a patient with ODD. The authors also reported macular microvascular attenuation only in the superficial capillary plexus. Although our patient had a visual field defect only in his left eye, reduced vascular density in both eyes was detected, especially in the nasal peripapillary area. Macular vascular density was found to be reduced in the deep capillary plexus layer as well as the superficial capillary plexus. It was interesting that the areas of nonperfusion were different in the superficial and deep capillary layers. This may be related to the superficial location of the ODD in the left eye, which caused less compression of the deep capillary layer.

In recent studies, OCTA imaging of the optic nerve head has revealed capillary narrowing in the superficial capillary plexus layer, areas of capillary dropout, and decreased vascular density in ODD patients.^8,16^ Cennamo et al.^17^ reported that ODD patients had lower flow index and reduced vascular density on optic nerve head OCTA compared to the control group. In addition, OCTA findings were positively correlated with ganglion cell layer thickness on OCT, and the authors emphasized that flow rate measurements made with OCTA may be an early predictor of axonal damage in ODD patients.^17^ Unlike in other studies, our patient showed decreased vascular density on macular OCTA as well as optic nerve head OCTA. Therefore, areas of reduced vascular density detected by OCTA may be a predictor of future central scotoma. These findings support the hypothesis that enlarged ODD may cause acute or chronic ischemia by compressing nerve fibers or surrounding vessels. The prominent hyperautofluorescence on FAF, visual field defect, and RNFL thinning in our patient were compatible with superficial drusen. Additionally, the superficial capillary plexus was more affected than the deep capillary plexus on macular OCTA due to the superficial location. Macular OCTA findings also supported the FAF, visual field, and OCT outcomes.

In conclusion, OCTA has become more widely used for the evaluation of optic nerve pathologies as well as retinal diseases because it is a noninvasive, easy, fast, and practical method. OCTA can be used as an auxiliary diagnostic method to USG and FAF in the diagnosis of ODD. Although ODD is generally asymptomatic, OCTA evaluation of the optic nerve head and macula may play an important role in the early detection of ischemic complications. This should be further investigated in prospective studies with long-term OCTA follow-up of ODD patients.
